# Hospital and Institutional News

**Published:** 1913-03-29

**Authors:** 


### HOSPITAL AND INSTITUTIONAL NEWS.

### YORK COUNTY HOSPITAL.

The *Leeds Mercury* of the 19th inst. contained the following official announcement, which it is stated had been issued to the press on the 18th, although a copy has not reached us:—

"The members of the House Committee and the Medical Board met at the hospital this morning to consider the imputations and charges made against this institution in the columns of The Hospital on the 15th inst.

"As the result of their investigations and the real facts disclosed, they were more than satisfied that there is a complete answer to the imputations and charges which have been so unfairly published against them; but they consider that the most satisfactory way to deal with the question, in order that the public may be reassured, apart from their own emphatic statement, is to obtain the appointment of some competent and independent authority to hold an inquiry into the whole of the allegations, and, as they have nothing to fear, it is proposed to place such result in the hands of the public. Accordingly they have made application to the President of the Royal College of Surgeons of England to appoint someone who has knowledge of hospital management, and is at the same time free from all local influence, and who will hold an impartial and independent inquiry. (Signed) G. Palmer, Hy. Ernest Leetham (Sheriff of York), John Bickle (President Workpeople's Hospital Fund), W. H. Jalland, F.R.C.S., and Peter MacDonald, M.D."

The above proposal cannot be deemed satisfactory, if, as we gather, it means *a private investigation* with closed doors. What the public, the patients and their friends, the hospital governors and the teaching schools, upon which latter the York County Hospital is dependent for its resident medical staff, are entitled to have, and must insist upon, is a public inquiry, to which everyone concerned will be called and at which the press will be invited to be present.

### INSURANCE PATIENTS IN REVOLT.

For the second time within a few weeks we have to record the revolt of sanatorium patients, this time from an institution in connection with the King Edward VII. National Memorial of Wales, now under the State Insurance scheme. The Udal Torre Sanatorium in South Devon has been abandoned by eleven patients, who have returned to their homes in South Wales. This return was a protest against the bad treatment which they allege to have received, and in particular, as is not infrequently the case with patients' complaints, against the food and the service, accusing the female staff of incivility and the administration of supplying not merely unappetising food but dishes used by some to be washed up by others. The porridge is stigmatised as poor and the ham as almost uncooked. These complaints, however, have been crystallised into a public statement by Mr. F. C. Morgan, of Cardiff, who appends the following Sunday programme as a sample of the system rather better than that which any week-day could show: "6.50 a.m.—Take temperature in bed. 7.0.—Rise, make bed, and get ready for breakfast. 8.0.—Breakfast, which consisted of porridge (on a cold plate), ½ oz. of butter, bread, and a plate of ham (practically raw). 9.15.—Cleaning ward. 10.50.—Exercise on the ground. 11.50 to 12.5.—Rest. 1.0.—Dinner—cold meat, pickles, and poor potatoes. 4.50.—Temperature taken. 5.5.—Cup of tea. 6.30.—One hard-boiled egg, ½ oz. butter, and bread, with coffee. 7.45.—Prayers. 8.30.—Lights out; strict silence." The medical superintendent, however, must be given a chance of equal publicity. We hope he will avail himself of it in our columns, and for the moment, therefore, refrain from comment.

### NEW SECRETARY FOR WALSALL HOSPITAL.

Mr. Frank Inch, assistant secretary to the King Edward VII.'s Hospital, Cardiff, has been appointed secretary to the Walsall and District Hospital in succession to Mr. Harold Wigg, who has taken up another appointment. The Walsall Hospital will greatly miss the valued services of Mr. Wigg, who has faithfully carried on the traditions of his predecessor—the first secretary of the Hospital, the late Mr. Samuel Welsh—but, from the reports we have received of Mr. Inch's training and qualifications, the choice of the new secretary should prove a very happy one. Mr. Inch commenced his hospital training in the Bristol Royal Hospital for Sick Children and Women, proceeding to Cardiff to a position in the secretary's office in November 1908. Upon the appointment of the then assistant, Mr. A. Ernest Wilkes, to the secretaryship of St. Mary's Hospital for Women and Children, Plaistow, Mr. Inch was at once promoted to this office. His loyalty and devotion to duty, his quick grasp of affairs, and the humanity and consideration which he has always displayed to the Cardiff patients and to those with whom he came in contact, have marked him out for certain promotion in the hospital world, and the hospital at Cardiff loses in him an exceptionally able and conscientious official.

### UNRULY BOYS IN CONVALESCENT WARDS.

A painful impression has been caused in Camberwell by the publication of a report, signed by Dr. Keats, the medical superintendent at the infirmary, on the punishment of unruly boys in the convalescent wards. Dr. Keats justly maintains that the rules of the infirmary and the directions of the staff must be implicitly obeyed, and that in the boys' wards, where between 60 and 70 lads, varying in age from five to sixteen years, are accommodated, it is difficult to maintain order, especially during the stage of convalescence. Like all boys, Dr. Keats' patients are full of mischief, but in hospitals and infirmaries order has been maintained hitherto without an appeal to corporal punishment. Dr. Keats in his report states that

In the last six months he has chastised four boys, ranging from seven to thirteen years old, by means of five strands of moderately thick string, the identical means which he used to correct his own child. He trusted the Guardians would approve of this method, adopted only after some years' careful consideration, as the best means of dealing with specially troublesome boys. The infirmary committee expressed approval of Dr. Keats' action, but the board decided to adjourn consideration of the report.

### INFIRMARY OR HOSPITAL: WHICH IS RIGHT?

With the idea of getting rid of the word "Infirmary" Major Chauncey moved, Councillor Aylmore seconded, and with an amendment from Colonel Wyndam, the governors of Chichester Infirmary have passed a resolution that their institution shall be known in future as The West Sussex Hospital. Apart from the inconvenient fact that certain possible subscribers still reject appeals on behalf of infirmaries on the ground that "these are already paid for out of the rates," there is a feeling that in practice the word hospital has been appropriated by the voluntary system, and that the word infirmary is the legitimate property of the Poor-Law institution. As this distinction roughly corresponds to reality, we think both Cardiff and Chichester, to take two recent examples, were wise in letting the name correspond to the theory. Both the voluntary institution and that maintained by the public purse have a proper pride in their respective titles. It is fitting that the voluntary hospitals should derive their title from the word guest-house, where a moiety of their cost alone is demanded of those who are received for treatment there. No smaller cause for pride resides in the Poor-Law infirmary, which derives its title from quarters set apart for the weak, those infirm members of society whom chance or change has compelled to seek refuge there. Each title represents, with the vividness of true etymology, the functions of the two types of institution, and the pride of the working-man subscriber to the voluntary hospitals is finely mirrored in the preference of some among the poor for treatment at the Poor-Law infirmary. Such a one is often heard to say, "I never subscribed a penny to the hospital; but I have been a ratepayer before now." It is as well that there should be no confusion, and that the false ideas that are sometimes still current as to the meaning of the two terms should be laid whenever a rechristening takes place.

### PORRIDGE AND MILK INSTEAD OF INSURANCE.

The latest criticism of compulsory insurance comes as a deduction from the important report on the diet of the labouring classes of Glasgow which has been published by the Physiological Department of Glasgow University. During 1911 and 1912 an inquiry has been in progress there conducted by Miss Dorothy E. Lindsay, B.Sc., Carnegie Research Fellow. The result is the present report, which is prefaced by an introduction by Dr. Noel Paton, Regius Professor of Physiology. After summarising the object of the investigation in the question, "Do the working classes of the city get such a diet as will enable them to develop into strong, healthy, energetic men and to do a strenuous day's work?" and, if so, is it obtainable cheaply enough to leave a margin for the expenses of family life plus something over for their pleasures "without which the very continuance of life is of doubtful value"? Dr. Paton proceeds to show that if this diet is not obtainable on these terms even a small compulsory levy for insurance may impoverish an already meagre diet, and its benefits may be bought too dearly. The deduction from this common-sense criticism is one that will startle our party politicians, for it means that compulsory feeding should precede compulsory insurance. The idealists who have preached the communisation of bread are therefore vindicated. "Give people food first and then necessarily see that they earn it" is thus shown to be the highest political wisdom. As has been pointed out, we insist on everyone, however poor, being provided with clothes, because nakedness is intolerable, and the State hastens to provide clothes for those without. Why should it not do so in the matter of food also? For the upshot of the report is that family life is unsupportable, in the scientific sense of the Physiological Department, on anything less than £1 a week, and even that is too small to guarantee a proper diet and the other things on which Dr. Paton rightly insists. A great many Glasgow families continue to subsist on less than this sum. The only palliative suggested is a partial return to the former national staple diet of Scotland, porridge and milk, in place of tea, bread and jam. But the above deduction shows the more excellent way. Diet need not be subject to risk; health must be. Therefore give compulsory food precedence over compulsory insurance, for in the last analysis food is the measure of physique.

### A HOSPITAL FOR SECTARIAN PROPAGANDA.

It seems almost incredible at this time of day that a hospital should be promoted for sectarian purposes, yet this is advanced "as one of the strongest arguments" in favour of a Jewish hospital by a Jewish contemporary. So much beside the mark is their argument, to grace a piece of sectarian propaganda by such a name, such stark ignorance does it betray of the hospital situation in the East End of London, that it is worth while as a matter of record to quote the published statements on which it rests. The "onslaught of missionaries," we learn, can be met by a Jewish hospital; and as the social functions of a certain association are held to have acted as "an effective counter-diversion" to similar attractions arranged by missionaries, so, we quote once more, "what is now being done in the sphere of social entertainment it is hoped, by means of the Jewish hospital, to accomplish in the domain of pain." After reading this nonsense, for that is what this is from the point of view of statesmanship and the hospital administration of London as a whole, we do not share our contemporary's wonder as to "what it is that makes so many who are prominent in the community hold aloof" from a project which, in the light of the above, has degenerated from the unwisdom of its beginning to sectarian propaganda of this romantic kind. Hospital administration, either in an isolated institution or in its co-ordinated aspect as a service for the Metropolis, has no room for parochialism of this temper. No scheme could survive such an attitude of mind in its promoters. This scheme has been already killed by it.

### MEDICAL CERTIFICATES IN IRELAND.

The question of the payment for medical certificates required by approved societies and Insurance Committees has been agitating the Irish members of the medical profession for some time. The Irish Insurance Commissioners have been in negotiation with the conjoint Committee of the British and Irish Medical Associations, with the result that the latter has agreed to one of the schemes of payment by capitation for certificates recommended by the Commissioners. The Exchequer grant of £50,000 is, subject to certain deductions, to be distributed on a capitation basis by the Insurance Committees among the panel practitioners, who undertake to provide all medical certificates required by approved societies and Insurance Committees. The country will be divided into four sub-divisions, with the respective capitation rates for medical certificates. The sub-divisions will be the six county boroughs and urban districts with a population of over 10,000; the most inaccessible areas; the counties with a net acreage per person of less than seven; and the counties with a net acreage per person of seven and upwards. Difficulties between doctor and patient may be referred to a committee of complaints consisting of two doctors, two representatives of the insured person, and a chairman chosen by the Insurance Committee. This body will report to the Insurance Committee, who, if either patient or doctor is at fault, may transfer the patient to another practitioner. If the practitioner is at fault, the committee cannot remove the practitioner from the panel or inflict any penalty upon him beyond the transfer of the insured person.

### OUR REPORT AND ITS SEQUEL AT SUNDERLAND

We publish this week a very interesting article in our "Eminent Chairman Series" on the work and personality of Mr. George Butchart, Chairman of the House Committee of Durham County and Sunderland Eye Infirmary. It is interesting to read of the more recent changes which he has done so much to introduce successfully in the light of our Report on the institution, which appeared on February 24, 1912. The accommodation for the house surgeon, the resident staff, and the nurses was then described as unworthy of the institution. As a direct outcome of the Report, and at the personal instigation of Mr. Butchart, the accommodation for the nurses, many of whom had to sleep out, has been considerably improved, and the committee have decided to enlarge the Eye Infirmary so as to obtain proper quarters for the house surgeon and nurses. Some difficulty has been found in obtaining suitable ground for building, but we understand that a scheme is now on foot, the prosecution of which is mainly due to the Chairman of the House Committee. It gives us very great pleasure to summarise the spade work which an energetic chairman and committee are now carrying on, for deeds are the best eulogy which any chairman and institution can have.

### FLORENCE NIGHTINGALE HOSPITAL EXTENSION.

Three years ago this admirable institution was re-opened in new premises at Lisson Grove, owing to the expiration in 1909 of the lease of the familiar building in Harley Street in which it flourished for almost sixty years. Now the remainder of the new site has become vacant, and by the terms of the lease must be built upon. Accordingly, a new wing has been begun which will add six more private rooms to the hospital and also extended quarters for the staff. The money for these extensions has been partly subscribed, but over £2,000 still remains to clear the new building of debt; and even then there is no endowment to provide for its upkeep. At present there are thirty-one beds in cubicles and private rooms, and eighteen patients waiting admission. As is well known, there is no charge for medical or surgical treatment in the hospital; even operations are performed absolutely free by the patients' own surgeons. And the charges for accommodation are so modest that they do not nearly cover the expenses of administration. This hospital does a great work by providing first-class nursing home accommodation for many ladies who shrink from a free hospital and yet cannot afford the serious expense of a private nursing home.

### A COMEDIAN'S THANKS.

Mr. A. Fletcher, the secretary of the subscriptions committee of the Newcastle Infirmary committee, has received from Mr. Dan Rolyat, the comedian, who was recently injured during a performance at the Tyne Theatre, Newcastle, a cheque for £68 7s. 6d., for the expenses of his nursing and maintenance, and a donation of fifty guineas. In an accompanying letter Mr. Rolyat says: "I am now on the high road to recovery; this happy state I owe to all those kindly and brilliant ladies and gentlemen under whose care I was placed and to whom I shall be for ever indebted." The hospitals owe so much to the various professions connected with the theatre, and especially perhaps to actors, that this latest instance of appreciation of care and treatment is doubly welcome. May the theatre and the hospital long continue to help each other in times of need!

### THE EXTERMINATION OF BIG GAME.

It is hardly surprising that Dr. Warrington Yorke's proposals for exterminating big game, on the assumption that they are the chief permanent reservoir of sleeping sickness, met with little sympathy from an audience at the Zoological Society, which included men whose interests are bound up with the preservation of animals of all kinds, such as Dr. Chalmers Mitchell, the curator of "The Zoo," and Mr. Walter Rothschild, to mention two only. Yet all that Dr. Yorke really appeared to urge was that a thoroughly scientific experiment should be undertaken in some particular district of the fly area to see what effect the extermination of the game would have on the spread of the disease. A good case can be made out for the preservation of the wild life, as well as the wild animals, by naturalists, zoologists, and scientists generally, but we have much less sympathy with the selfish and superficial claims of the mere sportsman.

### CAMP COOKERS DURING A STRIKE.

The trouble caused by the cutting off of the gas supply in Birmingham for thirteen out of the twenty-four hours and of the hydraulic water supply for the lifts during the coal strike, led to the invention of a very interesting substitute for gas-cooking at the General Hospital which should be suggestive to other institutions. Thanks to the kindness of Lieut.-Colonel E. Martineau, the camp cooker of the 6th Warwickshire Regiment was lent with the services of the Sergeant-Instructor. This, the new report records, met the difficulty of cooking for the patients without inconvenience, though the meal times of the resident staffs had to be altered. We are glad to publish this hint to other hospitals in case of future difficulty.

### A NEW HOSPITAL'S FIRST APPEAL.

The Lady Chichester Hospital for Women and Children, Brighton, which was opened last November to help those self-supporting gentlewomen who in time of sickness are ineligible for a free hospital, and who cannot afford the expense involved in going to a nursing home, has issued its first appeal. Accommodation is provided for twelve patients at fees varying from seven shillings and sixpence to three guineas, according to rooms. There are two private wards, and the hospital is entirely staffed by women. The President, Lady Chichester, and the Secretary, Miss Mabel Turner, of Gloucester Place, Brighton, unite in affirming that "it cannot be self-supporting" and appeal for annual subscriptions. The hospital has an out-patient department, and a branch at Hove, controlled by the same council but with its own executive, and is intended for cases of nervous breakdown. The invaluable work accomplished by the Florence Nightingale Hospital for Invalid Gentlewomen in Lisson Grove has, or ought to have, prejudiced the public in favour of a similar institution. A high standard has been set; there is an inspiring example to follow.

### NEW SECRETARY AT BOURNEMOUTH.

Mr. Gordon M. Saul, the assistant secretary at the Norfolk and Norwich Hospital, has received the appointment of secretary to the Royal Victoria and West Hants Hospitals at Bournemouth. He has been working under Mr. Frank G. Hazell, the secretary at Norwich, for the past five years, and has taken a keen interest in Friendly Society work, especially in its connection with hospitals. In view of the amalgamation scheme recently undertaken at Bournemouth, Mr. Saul takes up his post at a time of increased opportunity for useful work, and we congratulate him on his appointment.

### MORTUARIES FOR CORONERS' COURTS.

The important article which we publish this week on the mortuary unit appears with special significance from the fact that mortuaries in connection with Coroners' Courts are now occupying increased attention. A new departure, or rather, development, occurred four years ago, when the Herscher apparatus for preserving bodies by the agency of formalin was installed in the mortuary attached to the City Coroner's Court. It has proved of much value in identifying bodies. The City Coroner, Dr. Waldo, now urges the importance of a similar apparatus for Southwark, where many unidentified bodies are rescued from the Thames. A new mortuary, as Dr. Waldo points out, is really needed for Southwark, and this suggestion, in the light of the new special purpose for which the proposed Herscher apparatus would adapt it, shows again how varied are the uses, and consequently how careful should be the designing of mortuary units. We have followed up our repeated criticisms of the slackness with which mortuaries are still supervised in many institutions by this series of articles, which show what an example of forethought continental designers set our committees and architects in this respect. The possibilities suggested by a general adoption of the Herscher apparatus in mortuaries attached to Coroners' Courts shows the importance of a study of comparative design.

### A PATHOLOGIST'S DISPUTE.

Mr. George Leslie Eastes, M.B., of 38 New Cavendish Street, moved last week for an interlocutory injunction to restrain Mr. Charles Russ, M.B., M.R.C.S., of 25 Beaumont Street, from carrying on within ten miles from 62 Queen Anne Street, W., laboratory work similar to that which he had performed for Mr. Eastes under an agreement drawn in December 1905. Mr. Eastes' position was described by counsel as that of a pathologist, and that the above address was styled "The Laboratories of Pathology and Public Health." In 1905 he was carrying on the same profession at Queen Anne Street when the defendant was appointed assistant pathologist on the staff. Subsequently the defendant started practice as a pathologist on his own account at Beaumont Street, and this, the plaintiff contended, was a breach of the terms of the defendant's engagement under which he was "required to enter into a bond not to engage in similar work within a distance of ten miles from the laboratories, either for himself or on behalf of any other institute of a like character, under a penalty of £250." Mr. Justice Sargant directed that the dispute should be dealt with at the trial of the action. Till the result of this be known we, of course, refrain from comment.

### RECOMMENDS RESURRECTED IN MANCHESTER.

On the ground that it is not desirable that the number of waiting persons in the home district of Manchester Royal Infirmary should be increased while beds are occupied from distant neighbourhoods, which contribute next to nothing to the institution, the infirmary board has passed the following resolution:—"That persons resident elsewhere than in Manchester and Salford applying for admission as in-patients must lodge with the general superintendent a recommend signed by a trustee resident or carrying on business in the town or district within which the applicant resides; but the recommend may be dispensed with in exceptional cases with the consent of the general superintendent. This resolution shall not apply to cases of accident or emergency, which will be treated as heretofore." Sir William Cobbett explained that the recommendation system was formerly a means of obtaining the views of local medical men as the state and means of would-be patients. Lately the investigations of the infirmary staff and of the District Provident Association had largely replaced them, except for distant cases whose circumstances could not easily be examined locally. The present resolution is also a protest against the abuse of the infirmary funds, which results from distant districts, or particular firms close at hand for the matter of that, sending patients and withholding or refusing subscriptions.

### TRIBUTE TO A COTTAGE HOSPITAL SECRETARY.

The question of imposing, if the word may be allowed, a voluntary hospital levy on the fishing industries which are served by local hospitals has been mooted of late, but we are glad to note that Gorleston Cottage Hospital has received without such persuasion increased subscriptions from the fish merchants and fishing crews of Yarmouth and neighbourhood. The result is almost one hundred pounds in hand, though, we believe, Gorleston Hospital is one of those which benefited by the Payment of Members Act, since Mr. Arthur Fell, the local M.P., set an early example in handing over his superfluous £400 to the hospitals in his constituency. The worker who has been singled out for praise for this successful year is Mr. Rogers, who has thereby proved by his energy how very much the financial success of a cottage hospital depends upon the exertions and personality of the secretary. We should be glad to hear Mr. Rogers' views on the relation of the fishing industry to the local hospitals, and on the kite which has been flown for making a more systematic appeal to the sea-going craft of the neighbourhood.

### THE MALE STAFF IN LONDON HOSPITALS.

We have been asked what course hospital managers should take if, as is threatened, an attempt is made by the officers of the London County Council to impose for the first time a tax upon the male staff employed in voluntary hospitals. The working male staff consists of messengers and attendants, and no doubt for this reason the Government have never attempted to impose a tax upon any such employee in a hospital. The definitions on the Form for England and Wales under the head of "Male Servants" state clearly that this term does not include a servant who, being *bona-fide* employed in some other capacity, is occasionally or partially employed in any capacity involving the duties of any of the descriptions of servants included in Definition 1. The only male servant mentioned in the definition that could be arguable is the house porter, but in the voluntary hospitals the man denominated house porter is in practice the gate porter more often than not, and that officer is not in any way employed in any of the duties of servants liable to taxation. The male staff mainly consists of messengers and attendants who do in fact come under the "Exemption of Male Servants" set out in Number 2 of the definitions on page 3 of the form. This is so because such messengers and attendants fulfil the conditions of exemption which are: persons who "perform non-taxable work as their substantial employment" and "taxable work in a minor degree only, if at all." It is a very grave matter indeed that there seems to be a dead-set at the present time against hospitals by representatives of the Government and local taxation authorities, who seem never to fail to try to impose additional cost on the voluntary hospitals, notwithstanding the managers of those hospitals, by their public spirit and generosity in relation to the National Insurance Act, have become in fact its mainstay.

### SUICIDE OF A WORKHOUSE CHAPLAIN.

We regret to record the death of the Rev. James Alfred Nash, chaplain at St. Marylebone Workhouse, who was found shot in his bed by his landlady at his rooms at Paddington Green on Good Friday morning. The inquest mainly substantiated the reports of the affair, which stated that the landlady heard the sound of a shot, entered his room and found Mr. Nash with a bullet wound in his head and an exploded revolver in his right hand. By his side was a tumbler containing potassium cyanide. Mr. Nash, who had studied at the London College of Divinity, was ordained in 1877 to the curacy of Clerkenwell, which he held for seven years. St. Saviour's, Fitzroy Square, where he stayed a year only, was succeeded by the curacy of St. Barnabas, Marylebone, which he held until he was appointed chaplain at St. Marylebone Infirmary in 1900. From evidence given at the inquest it appeared that Mr. Nash had often expressed exaggerated fears of being run over by motor omnibuses, and that he constantly reminded an undertaker of his acquaintance of his wish to be cremated. He bought the cyanide seemingly for photographic purposes, and was apparently much worried about a boy in whom he was interested, who had been charged with theft. The verdict was suicide while of unsound mind.

### A DISTINGUISHED RIVIERA FIGURE.

Among the names which stand out of English physicians who have gained a great reputation among their fellow-countrymen abroad is that of Dr. Philip Frank, who has just died at the age of eighty-three. He was educated partly in England and partly in North Germany, and he graduated M.D. at Berlin with distinction. He then started his travels by joining the Army Medical Service, when he visited South Africa, and finally became travelling physician to Lord Brownlow. On his friend's death, for Lord Brownlow was quite as much friend as patient, Dr. Frank took up his residence at Cannes, where his reputation attracted not merely patients among the foreign colony, but the respect of his French colleagues. Among the former were Prince Ferdinand and Princess Christian, and the present King of Bulgaria; while his abilities as an ophthalmologist attracted many, including the poor, from great distances. He was head of the ambulance at Balan during the siege of Sedan, but after the war was over he returned to Cannes. The excess of work induced him at last to retire from practice. By his death one of the most noted figures on the Riviera passes away, and now that the season is at its height it is true to say that his loss is felt throughout the whole district.

### SELLING STOCK OR CHARGING PATIENTS?

Earnest discussion on a resolution authorising the trustees of the Worcester General Infirmary to sell stock to produce not more that £5,000 forced the above question on the annual meeting last week. The alternative to the suggested sale was that patients should be asked to contribute three shillings and sixpence per week. The suggestion at first sound appears repellent, but no hasty judgment must be formed, since a system previously described in The Hospital on similar lines has been inaugurated with success at King Edward VII.'s Hospital, Cardiff. The resolution, however, was carried.

### DIPHTHERIA IN COVENTRY HOSPITAL.

An outbreak of diphtheria was reported to the General Committee of the Coventry and Warwickshire Hospital last week, and in consequence the hospital has been closed to visitors. Mr. W. J. Wormell stated that four cases had been isolated at the hospital, that others had been placed in the City Hospital, and that the cause of the outbreak is being investigated. A resolution, moved by Mr. Hill, was unanimously carried, urging the city authorities to provide for the treatment of diphtheria cases.

### A HOSPITAL FOR MANXMEN.

The first annual meeting in the new institution, which Lord Raglan opened last September, for the Noble's Isle of Man Hospital was a scene of considerable enthusiasm. The Manxmen are naturally proud of the fact that they have no longer to "go across the water" for hospital treatment. For the second year the income has exceeded the expenditure, but, as the Chairman wisely pointed out, against this must be set the momentary absence of expenditure for repairs, which the occupation of a new building at first obviates. The money, however, has been ear-marked as a kind of sinking fund. Mr. R. D. Gelling has been re-elected honorary secretary, when a tribute was paid to the experience which he has acquired during the years that he has held that post. It is, perhaps, as well to remind English readers that Noble's Isle of Man Hospital is not at Douglas but an insular institution. A rather original function took place at the annual meeting when two clocks, the gifts of patients in appreciation for the treatment which they had received, were unveiled by Lady Raglan. Dr. Pantin, a member of the staff, is understood to have started the idea of enlisting the sympathy of past patients, whose gratitude is commemorated in both corridors, where the clocks have been placed.

### THE WARD FLY NUISANCE.

The only impression that the present writer still retains of his first visit to a hospital ward is of a typhoid fever case, and of a nurse kept constantly busy fanning away some persistent flies which tried to settle on the patient's face. With the approach of summer, especially after so mild a winter as we have had, means of minimising the fly nuisance are well worth consideration by hospital managers. For in hospital, even more obviously than elsewhere, the presence of flies is a danger. There is no doubt that by efficient fly-killing measures the number of these insects in any given institution can be vastly reduced, though attention has also to be paid to the destruction of their breeding places anywhere in the vicinity, such as heaps of garbage, manure, and the like. In the United States Government Departments do not think it beneath their dignity to take the matter up; and the State entomologist of Minnesota has designed a fly-trap of cheap and simple construction which has proved itself capable of catching twelve thousand flies a day in a heavily infested region. Some fresh bread and milk is placed in a pan at the base of the trap, and access to it is through a rather twisty passage. Above the bait is an easy broad passage into a space from which exit is almost beyond fly ingenuity; this space is enclosed by fine wire meshwork of the meat-safe order, and boiling water can be poured all over it every now and then to kill the imprisoned flies. The idea is an old one, but attention to detail has improved it, and the trap may be recommended in advance to hospitals and institutions.

### THIS WEEK'S DRUG MARKET.

The value of cod-liver oil is well-maintained, but the advancing tendency has been checked somewhat, although no improvement in the catch of cod or the yield of oil is reported from the Norwegian fishing grounds. The catch of cod up to the middle of the month was only little more than a third of that at the same date last year, and the yield of oil up to the middle of the present month was about ten thousand hectolitres against twenty-nine thousand at the same date last year; these statistics certainly support the view that prices are more likely to advance than to recede. Business in opium is extremely quiet and there is no change in the position. Carbolic acid appears to be tending somewhat cheaper. At the public sales in Mincing Lane Cape aloes realised higher rates, orange peel was slightly dearer, and sarsaparilla rather lower. Business has naturally been quiet since last report appeared as the result of the Easter holidays.

